# Species inventory and morphological measurements of spiders (Arachnida, Araneae) and ants (Insecta, Hymenoptera, Formicidae) collected in northern Ghana

**DOI:** 10.3897/BDJ.14.e163267

**Published:** 2026-01-07

**Authors:** Veikko Yrjölä, Luís C Crespo, Stephanie Saussure, Francis B Asamoah, Arttu Soukainen, Benjamin K Badii, Pasi Sihvonen, Pedro Cardoso

**Affiliations:** 1 LIBRe - Laboratory for Integrative Biodiversity Research, Finnish Museum of Natural History, University of Helsinki, Helsinki, Finland LIBRe - Laboratory for Integrative Biodiversity Research, Finnish Museum of Natural History, University of Helsinki Helsinki Finland; 2 University of the Azores, CE3C—Centre for Ecology, Evolution and Environmental Changes, Azorean Biodiversity Group, CHANGE—Global Change and Sustainability Institute, School of Agricultural and Environmental Sciences, Ponta Delgada, Portugal University of the Azores, CE3C—Centre for Ecology, Evolution and Environmental Changes, Azorean Biodiversity Group, CHANGE—Global Change and Sustainability Institute, School of Agricultural and Environmental Sciences Ponta Delgada Portugal; 3 Natural Resources Institute Finland LUKE, Jokioinen, Finland Natural Resources Institute Finland LUKE Jokioinen Finland; 4 Department of Ecotourism, Recreation and Hospitality, University of Energy and Natural Resources, Sunyani, Ghana Department of Ecotourism, Recreation and Hospitality, University of Energy and Natural Resources Sunyani Ghana; 5 Department of Wildlife and Range Management, Kwame Nkrumah University of Science and Technology, Sunyani, Ghana Department of Wildlife and Range Management, Kwame Nkrumah University of Science and Technology Sunyani Ghana; 6 University for Development Studies, Tamale, Ghana University for Development Studies Tamale Ghana; 7 Finnish Museum of Natural History, University of Helsinki, Helsinki, Finland Finnish Museum of Natural History, University of Helsinki Helsinki Finland; 8 Centre for Ecology, Evolution and Environmental Changes (cE3c), Global Change and Sustainability Institute (CHANGE), Faculdade de Ciências, Universidade de Lisboa, Lisboa, Portugal Centre for Ecology, Evolution and Environmental Changes (cE3c), Global Change and Sustainability Institute (CHANGE), Faculdade de Ciências, Universidade de Lisboa Lisboa Portugal

**Keywords:** agriculture, biodiversity, checklist, mango orchard, morphometrics, standardised sampling, West Africa, West Sudanian savannah

## Abstract

**Background:**

Agricultural expansion, a leading driver of biodiversity loss, has widespread effects on ecosystem services, particularly in tropical regions. In West Africa, the impact of intensified agriculture on local biodiversity – especially predator and decomposer species like spiders and ants – is understudied. This study aims to provide a checklist of terrestrial spiders and ants associated with savannahs and mango orchards in northern Ghana thus creating a baseline for further ecological studies on the community composition of these groups.

**New information:**

In this data paper, we publish the baseline checklist and morphological measurements of spiders (Araneae) and ants (Hymenoptera, Formicidae) associated with forest savannahs and mango orchards located in northern Ghana. In total, we collected 64 species (28 unidentified morphospecies) of spiders and 64 species (24 unidentified morphospecies) of ants. Of these, almost all spider species and nine ant species were new records for Ghana, while many of the morphospecies could potentially be species new to science. In addition, we publish standardised morphological measurements of each species for potential functional diversity studies in the future.

## Introduction

Biodiversity loss – including decreasing genetic variability, population abundances and species richness – lowers an ecosystem’s capacity to maintain natural processes and its ability to provide goods and services to satisfy human needs. Despite our deep dependency on biodiversity (Cardinale et al. 2012), we are losing it alarmingly fast. One of the main drivers of biodiversity loss is agriculture (Dudley and Alexander 2017), as it leads to the conversion of natural habitats to managed systems, which, in addition to habitat loss, increases pollution, such as greenhouse gases and pesticide use. These factors are amongst the most important threats to spiders (Branco and Cardoso 2020) and insects (Mauda et al. 2018, Miličić et al. 2021) worldwide. By industrialising agriculture, we become even more dependent on a few crop and livestock species, while contributing to the mass extinction of others. For instance, richness and abundance of predator and decomposer arthropods have been shown to decline because of increased intensive agricultural practices (Attwood et al. 2008, Prieto-Benítez and Méndez 2011, Potapov et al. 2020, Melo et al. 2021). While most of the biodiversity-ecosystem functioning studies come from Europe, North America and Australia (Carrick and Forsythe 2020), similar studies need to be extended to all agricultural regions of the world.

The project "Sustainable intensification of food production through resilient farming systems in West and North Africa (SustInAfrica)" is a Horizon 2020 EU-funded capacity-building project targeting West and North African smallholder farmers to facilitate sustainable intensification of African farming systems. One of its objectives is to test and assess sustainable and resilient farming methods for mango (*Mangifera indica* L.), which, amongst other economical and environmental challenges, is losing significant yields to pest insects, such as the mango seed weevil (Coleoptera, *Sternochetus mangiferae* (Fabricius, 1775)) and mango fruit flies (Diptera, *Bactrocera* spp., *Ceratitis* spp.). In Ghana, farmers still mostly rely on the use of synthetic pesticides (Akotsen-Mensah et al. 2017), which may have both direct and indirect negative effects on non-target organisms and more sustainable agroecological practices promoting biodiversity and ecosystem services are needed. As a consequence, local biodiversity and its service providers (e.g. biological control agents) must be surveyed and monitored in order to evaluate the loss of ecosystem functioning due to farming.

We collected, quantified and identified spiders (Araneae) and ants (Hymenoptera, Formicidae) from two human-transformed mango orchards and one savannah habitat near Tamale, northern Ghana. The savannah sampling serves as a reference condition needed to quantify ecosystem integrity, i.e. to answer the question of how much different communities in agricultural areas compare with the original landscape dominated by savannah. Only with a baseline is it possible to know how much we lose in terms of taxonomic and functional diversity when relying on potentially adverse agricultural practices. Spiders and ants were chosen as study taxa because: 1) they are abundant and common in terrestrial ecosystems; 2) they provide many ecosystem services and disservices (Del Toro et al. 2012, Michalko et al. 2018, Cardoso et al. 2025) and 3) they have potential as bioindicators of ecosystem service provision (e.g. Gerlach et al. (2013)).

Numerous studies have been published on West African spiders since the late 19^th^ century. The earliest were pioneer expedition reports (such as Karsch (1879) or Marx (1893), to cite but two), but eventually more focused and specialised works appeared, such as those by Millot in the 1940s (Berland and Millot 1940, Berland and Millot 1941, Millot 1941, Millot 1942, Millot 1946) or those by Jézéquel in the 1960s (Jézéquel 1964a, Jézéquel 1964b, Jézéquel 1964c, Jézéquel 1965, Jézéquel 1966). As a result of the interest of taxonomists and the availability of large collections that allow for comprehensive taxonomic and systematic studies, the studies published have been largely biased towards certain groups. For example, the current knowledge of West African Ctenidae is significantly enriched after the works coordinated by Rudy Jocqué (Jocqué and Steyn 1997, Steyn et al. 2003, Jocqué et al. 2006, Henrard and Jocqué 2017), but other groups, such as the genus *Oxyopes* Latreille, 1804, an ubiquitous presence at African grasslands and shrublands, still lack a comprehensive revision of the African species that could allow proper identification.

In myrmecology, early notable authors include Gustav Mayr, Filippo Silvestri, Felix Santschi, Henri Stitz, Carlo Emery and William Morton Wheeler, who contributed to early ant taxonomy, including African species (https://antwiki.org/wiki/World_Ant_Taxonomists). Later, Barry Bolton became known for extensive taxonomic work on African ants, publishing identification keys and descriptions still used today (Fisher and Bolton 2016). More recent studies of ant diversity in West Africa were predominantly focused on ant assemblages in tropical forest-savannah (Belshaw and Bolton 1994, Yeo et al. 2011, Kaiser et al. 2015, Yeo et al. 2017), cocoa (Room 1971, Majer 1972, Kone et al. 2014) and mango orchards (Diamé et al. 2017, Taylor et al. 2018), to name but a few. The most recent study from north-western Ghana (Sosiak et al. 2024) recorded regionally unique ant assemblages across three habitats (floodplain, Guinea savannah and riparian forest habitats), protected within the Wechiau Community Hippo Sanctuary (WCHS). The authors found that the WCHS ant assemblage was relatively unique, sharing only about 35% of species found in similar Côte d'Ivoire habitats and 25% of other Ghanaian assemblages. As of 2024, there are 428 native ant species recorded from Ghana (antsmaps.org). Nevertheless, the surveys in Ghana and West Africa in general remain scarce and scattered and a thorough region-wide revision is needed.

In order to study the biodiversity of mango agroecosystems, it is essential to have comparable and reliable metrics. Metrics such as species richness and evenness, being widely used, measure the composition and structure of communities (Gaston and Spicer 2013). In addition to taxonomic identities, a functional dimension can be applied by collecting species traits (Wong et al. 2018). The selected traits — such as body length, fang length and eye position — are ecologically meaningful because morphological features often determine how insects and arachnids exploit resources and contribute to ecosystem functions, including pollination, predation and pest regulation (Macías-Hernández et al. 2020, Drager et al. 2023). By linking morphology to functional roles, these measurements provide insights into the ecological strategies and potential ecosystem services of the studied species (de Bello et al. 2010). Finally, changes in the functional composition of species assemblages can be related to losses of ecosystem function (Mouillot et al. 2013).

## General description

### Purpose

"SustInAfrica" is a research project targeting West and North African smallholder farmers to facilitate sustainable intensification of African farming systems. Its overall objective is to develop and deploy a reference framework on best agricultural practices and technologies, based on a systems approach and successfully verify their efficacy to intensify primary production in a self-sufficient, sustainable and resilient manner. With this work, we intend to present a comprehensive database for species and traits of both spiders and ants sampled in the region of Tamale, northern Ghana. This work contributes to the objectives of SustInAfrica as a baseline for future monitoring of spider and ant taxonomic and functional diversity as providers of important ecosystem services and disservices, such as pest control and herbivory.

## Project description

### Title

Sustainable intensification of food production through resilient farming systems in West and North Africa (SustInAfrica)

### Funding

SustInAfrica is funded by the EU Horizon 2020 Research and Innovation Programme under Grant Agreement 861924.

## Sampling methods

### Study extent

**Figure 1.**
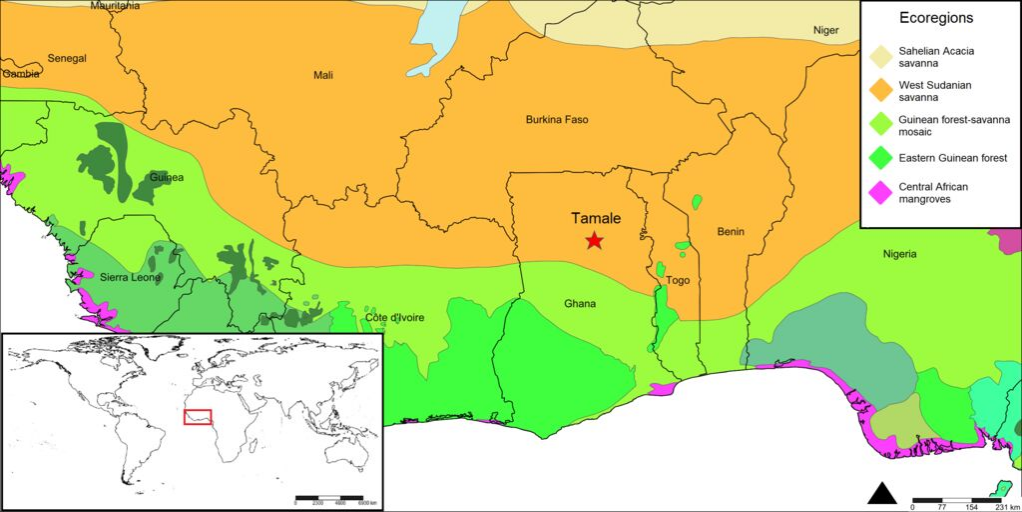
The research took place in the vicinity of Tamale (red star), a northern Ghanaian town that is located in the southern part of the West Sudanian savannah ecoregion (in orange colour). Map made with SimpleMappr.

The West Sudanian savannah, situated in West Africa, is a tropical savannah ecoregion. In the northern part of Ghana (Fig. 1), it encompasses a hot and dry wooded savannah, characterised by large tree species and extensive "elephant" grass. This habitat has faced significant reduction, degradation and fragmentation due to agricultural activities, fire and clearance for wood and charcoal (One Earth 2024). Additionally, overhunting has led to a drastic decline in the populations of many larger mammal species. The ecoregion, predominantly flat and ranging between 200 and 400 metres in elevation, lacks prominent topographical features. Its climate is tropical, with mean monthly maximum temperatures fluctuating between 30°C and 33°C and mean minimum temperatures ranging from 18°C to 21°C. Annual rainfall reaches up to 1,000 mm in the southern region, but decreases towards the north, with only 600 mm along the border with the Sahelian Acacia Savannah ecoregion. Rainfall is highly seasonal and the dry season can persist for several months.

### Sampling description

**Figure 2.**
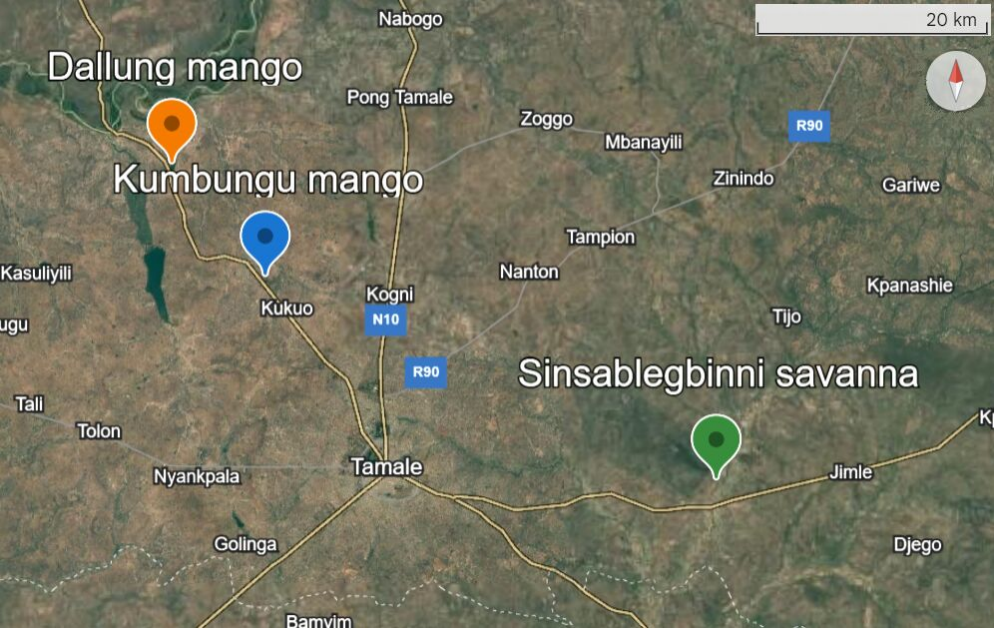
Localities of the study sites on a base map from Google Earth.

Sampling took place in three localities near the town of Tamale, northern Ghana (Fig. 2). In Kumbungu, a small town 20 km NW of Tamale (9°32'43.9"N 0°56'06.9"W), five 30 ✕ 30 m plots were each sampled with 32 pitfall traps and four Malaise traps. These plots were further divided into four approx. 10 ✕ 10 m subplots with eight pitfall traps and one Malaise trap. In Kumbungu, pitfall traps were grouped by subplot, with eigth traps combined into a single sample. In Dallung, a small town 30 km NW of Tamale (9°37'57.8"N 1°00'27.1"W), a smaller 10 ✕ 10 m plot was sampled with eight pitfall traps and one Malaise trap, with each pitfall trap treated as an individual sample. In addition to these two mango orchards, a partly forested savannah in the Sinsablegbinni Forest Reserve, located 25 km E of Tamale (9°23'26.5"N 0°36'01.4"W), was sampled with four 50 ✕ 50 m plots, each with 48 pitfall traps and one Malaise trap. In the forest plots, pitfall traps were grouped into sets of four per sample, yielding 12 samples per plot.

One pitfall trap consisted of a 350-ml plastic cup (80 mm diameter) with propylene glycol up to half depth and a paper sheet to cover circa 2 cm above the ground. The traps were situated at least five metres apart from each other along the plot borders. Malaise traps were 120 ✕ 100 ✕ 150 cm, Townes style, in white colour (Ento Sphynx, ref: 26.61). The Malaise traps were situated in the middle of each plot, usually in between two trees. The trapping interval lasted for fourteen days during October and November 2022. Due to flooding, some of the fieldwork was postponed in Kumbungu for two weeks (see Temporal Coverage for details). After sampling, the material was stored in 80% ethanol and transported to the Finnish Museum of Natural History (Luomus), University of Helsinki, Finland, for further processing.

### Step description

Spiders and ants were sorted from the bulk material. All of the spider specimens were first sorted into adults and juveniles and the adult specimens were identified into species and morphospecies depending on the available literature. Next, a maximum of five males and five females of each (morpho)species were measured for morphological traits. These same specimens were incorporated into the Luomus collections, the remaining specimens being shipped back to Ghana and are available at the collection of the University of Development Studies, Tamale, Ghana.

For ants, similar methods were used, except that not all identified specimens were separated from the samples after counting to save time and space. Due to the heavily female-biased sex ratio and high size variation, five to ten workers of each (morpho)species were measured for morphological traits and a maximum of ten individuals were stored in the Luomus collections, while the remaining specimens were shipped back to Ghana and are available at the collections of the University of Development Studies, Tamale, Ghana.

Leica S8AP0 and Leica M165C microscopes with Leica CLS 100 LED light sources were used for identification and morphometrics. Identification of higher taxonomy (families, subfamilies and genera) followed the literature of African spiders: an identification manual (Dippenaar-Schoeman and Jocqué 1997) and Ants of Africa and Madagascar: a guide to genera (Fisher and Bolton 2016). Identification to species was made by searching taxonomic descriptions and keys from online databases (spiders: World Spider Catalog; ants: Antwiki and Ants of Africa).

## Geographic coverage

### Description

Northern Region, Ghana, West Africa

### Coordinates

9.3793 and 9.6289 Latitude; −1.0077 and −0.5958 Longitude.

## Taxonomic coverage

### Description

The following taxa of the phylum Arthropoda are covered

### Taxa included

**Table:** 

Rank	Scientific Name	
phylum	Arthropoda	
class	Arachnida	
order	Araneae	
class	Insecta	
order	Hymenoptera	
family	Formicidae	

## Traits coverage

**Spiders**

1. Body size - Mean body length of adults. Spiders were measured from the closest point to the chelicera in the prosoma to the end of the abdomen (excluding abdomen appendages). Body size is consistently found to increase with temperature (e.g., Gibb et al. 2014), reflecting changes in metabolic rates. Gibb & Parr (Gibb and Parr 2013) also found that body size decreases with increasing habitat complexity for other arthropods. Finally, body size often mediates species’ responses to environmental change (Purvis et al. 2000, Chichorro et al. 2022a, Chichorro et al. 2022b, Oyarzabal et al. 2024).

2. Prosoma length - Mean prosoma length of adults, excluding chelicerae. Spider prosomas were measured at their longest. As body length is much dependent on abdomen size and this might change considerably depending on recent feeding or gravidity, often prosoma length is a more consistent measure depicting body size.

3. Prosoma width - Mean prosoma width of adults. Spider prosomas were measured at their widest. Moya-Larano (Moya-Laraño et al. 2008) proposes that prosoma width is a better measure of overall size than length. Other arthropods are narrower in more complex habitats (e.g., Sarty et al. 2006, Barton et al. 2011).

4. Prosoma height - Mean prosoma height of adults. Spider prosomas measured at their highest, including sternum. Prosoma height reflects the development of muscles and other internal structures, which might be important for hunting strategy, besides running behavior.

5. Tibia I length - Mean length of left tibia I of adults. Measured from lateral view at the mid-point of height. Tibia I length is related with running speed (Moya-Laraño et al. 2008). Might decrease intra-specifically with latitude because resource allocation for leg development is reduced with a shorter development time (Baba et al. 2012). Finally , limb length is expected to be larger in less complex habitats, as has been shown for other arthropod groups (Gibb et al. 2014).

6. Fang length - Mean fang length of adults. Fangs were measured from the base to the tip in a straight line. In orb-weavers, fang length is positively correlated with the speed of prey (Olive 1980, Olive 1981) and prey in warmer environments is expected to be faster-moving. Was also found to be related with elevation, latitude and vegetation density by Gibb et al. (Gibb et al. 2014).

**Ants**

Drager et al. (Drager et al. 2023) found overall body size, relative eye position and scape length to be informative for predicting diet/trophic position in ant communities. Specifically, trophic position was negatively correlated with body size and positively correlated with sensory traits (higher eye position and scape length).

1. Body size - Mean body length of worker caste. With body in lateral view, the sum of the length of the left mandible, head capsule, WL (= Weber's length), petiole and postpetiole (when present), and gaster. The body size has been

2. Head length - Mean head length of worker caste. With head in dorsal view, the length of a straight line drawn across the head of the ant at its longest point, including lobes but excluding spines and mandibles.

3. Scape length - Mean scape (first antennal segment) length of worker caste. The square root of the sum of the squared length of the left or right scape in dorsal view and the squared height of the scape with either the head in full face view or the body in lateral view, depending on the position of the antennae and of the ant on the point.

4. Eye distance - Mean distance between the compound eye and mandibular base. Shortest distance from the anterior most margin of the left compound eye in lateral view. Note, the eye position (EP) mentioned by Drager et al. is a ratio between the Eye distance and the Head length (ED:HL).

## Temporal coverage

**Data range:** 2022-10-06 – 2022-11-14.

### Notes

6-19 Oct 2022 (Kumbungu plots 1-3)

7-21 Oct 2022 (Sinsablegbinni plots 3-4)

8-22 Oct 2022 (Sinsablegbinni plots 1-2)

13-27 Oct 2022 (Dallung)

31 Oct - 14 Nov 2022 (Kumbungu plots 4-5)

## Collection data

### Collection name

Finnish Museum of Natural History (MZH): Hymenoptera World (Luomus), Arachnida and Myriapoda (Luomus)

### Collection identifier

http://tun.fi/HR.23, http://tun.fi/HR.46

### Specimen preservation method

Ethanol 96 %

### Curatorial unit

The Finnish Museum of Natural History (MZH), University of Helsinki, Finland

## Usage licence

### Usage licence

Open Data Commons Attribution License

### IP rights notes

CC-BY 4.0

## Data resources

### Data package title

Occurrences and morphological measurements of spiders and ants collected in Ghana, West Africa

### Resource link

https://doi.org/10.15468/h9vtau https://doi.org/10.5061/dryad.h18931zwc

### Number of data sets

3

### Data set 1.

#### Data set name

Occurrence data of spiders (Araneae) and ants (Hymenoptera, Formicidae) collected from northern Ghanaian mango orchards and forest savannah

#### Download URL

https://ipt.laji.fi/archive.do?r=osang

#### Description

The dataset contains occurrence data of 36 species and 28 morphospecies of spiders (Araneae) and 40 species and 24 morphospecies of ants (Formicidae) collected from ten sampling points in northern Ghana during Oct-Nov 2022. The total sample size is 288 samples of spiders with 438 mature individuals and 782 samples of ants with 7859 individuals. The dataset contains collection information (locality, habitat, collecting method, time, coordinates, collectors etc.) and sample information (count, sex, remarks etc.).

**Data set 1.** 

Column label	Column description
occurrenceID	An identifier for the dwc:Occurrence (e.g. Dallung-Plot-1-Sample-1-Spider-1).
locationID	An identifier for the set of dcterms:Location information (e.g. Dallung-Plot-1).
eventDate	The date-time or interval during which a dwc:Event occurred (e.g. 2022-10-13/2022-10-27).
samplingProtocol	The names of, references to, or descriptions of the methods or protocols used during a dwc:Event (e.g. 8 pitfalls).
family	The full scientific name of the family in which the dwc:Taxon is classified (e.g. Lycosidae).
subfamily	The full scientific name of the subfamily in which the dwc:Taxon is classified (e.g. Formicinae).
genus	The full scientific name of the genus in which the dwc:Taxon is classified (e.g. Trochosa).
scientificName	The full scientific name, with authorship and date information if known (e.g. *Myrmarachne kiboschensis* Lessert, 1925).
taxonRank	The taxonomic rank of the most specific name in the dwc:scientificName (e.g. genus).
identificationID	An identifier for the set of dwc:Taxon information (e.g. Thomisidae008_GHA).
identificationRemarks	Comments or notes about the dwc:Identification (e.g. "Could be A. wilsoni which occurs in South Africa").
identifiedBy	A list (concatenated and separated) of names of people, groups or organisations who assigned the dwc:Taxon to the subject (e.g. Luís Crespo).
dateIdentified	The date on which the subject was determined as representing the dwc:Taxon (e.g. 2023-03-17).
sex	The sex of the biological individual(s) represented in the dwc:Occurrence (e.g. female).
caste	Categorisation of individuals for eusocial species (e.g. worker).
individualCount	The number of individuals present at the time of the dwc:Occurrence (e.g. 1).
basisOfRecord	The specific nature of the data record (e.g. HumanObservation).
occurrenceStatus	A statement about the presence or absence of a dwc:Taxon at a dcterms:Location (e.g. present).
organismRemarks	Comments or notes about the dwc:Organism instance (e.g. "Missing two legs").
dynamicProperties	A list of additional measurements, facts, characteristics or assertions about the record. Meant to provide a mechanism for structured content (e.g.{"HW":0.4875,"HL":0.625,"CI":78,"SIL":52}; abbreviations were retrieved from "antwiki Mophological Measurements").
occurrenceRemarks	Comments or notes about the dwc:Occurrence (e.g. a new species).
continent	The name of the continent in which the dcterms:Location occurs (e.g. Africa).
higherGeography	A list (concatenated and separated) of geographic names less specific than the information captured in the dwc:locality term (e.g. West Africa).
country	The name of the country or major administrative unit in which the dcterms:Location occurs (e.g. Ghana).
stateProvince	The name of the next smaller administrative region than country (state, province, canton, department, region etc.) in which the dcterms:Location occurs (e.g. Northern region).
locality	The specific description of the place (e.g. 30 km NW of Tamale).
habitat	A category or description of the habitat in which the dwc:Event occurred (e.g. West Sudanian savannah).
decimalLatitude	The geographic latitude (in decimal degrees, using the spatial reference system given in dwc:geodeticDatum) of the geographic centre of a dcterms:Location. Positive values are north of the Equator, negative values are south of it (e.g. 9.544).
decimalLongitude	The geographic longitude (in decimal degrees, using the spatial reference system given in dwc:geodeticDatum) of the geographic centre of a dcterms:Location. Positive values are east of the Greenwich Meridian, negative values are west of it (e.g. −0.936).
geodeticDatum	The ellipsoid, geodetic datum or spatial reference system (SRS), upon which the geographic coordinates given in dwc:decimalLatitude and dwc:decimalLongitude are based (e.g. WGS84).
georeferenceSources	A list (concatenated and separated) of maps, gazetteers or other resources used to georeference the dcterms:Location, described specifically enough to allow anyone in the future to use the same resources (e.g. Google maps).
coordinateUncertaintyInMetres	The horizontal distance (in metres) from the given dwc:decimalLatitude and dwc:decimalLongitude describing the smallest circle containing the whole of the dcterms:Location (e.g. 300).
recordedBy	A list (concatenated and separated) of names of people, groups or organisations responsible for recording the original dwc:Occurrence (e.g. Francis Asamoah | Pedro Cardoso | Stéphanie Saussure | Arttu Soukainen | Fuseini Wumdei).
associatedOccurrences	A list (concatenated and separated) of identifiers of other dwc:Occurrence records and their associations to this dwc:Occurrence (e.g. id.luomus.fi/HV.25869).
countryCode	The standard code for the country in which the dcterms:Location occurs (e.g. GH).
kingdom	The full scientific name of the kingdom in which the dwc:Taxon is classified (e.g. Animalia).
id	ID of the data row added by the IPT (here same as occurrenceID).

### Data set 2.

#### Data set name

Morphological measurements of spiders (Araneae) collected from northern Ghanaian mango orchards and forest savannah

#### Download URL

https://datadryad.org/stash/dataset/doi: 10.5061/dryad.h18931zwc

#### Description

This dataset contains the values of morphological traits measured from mature spiders collected in northern Ghana October/November 2022. The six measured morphological traits for spiders are Total body length (mm), Prosoma length (mm), Prosoma width (mm), Prosoma height (mm), Tibia I length (mm) and Fang length (mm).

**Data set 2.** 

Column label	Column description
organismID	An identifier for the dwc:Organism instance.
family	The full scientific name of the family in which the dwc:Taxon is classified (e.g. Lycosidae).
genus	The full scientific name of the genus in which the dwc:Taxon is classified (e.g. Trochosa).
scientificName	The full scientific name, with authorship and date information if known (e.g. *Myrmarachne kiboschensis* Lessert, 1925).
taxonRank	The taxonomic rank of the most specific name in the dwc:scientificName (e.g. species).
identificationID	An identifier for the set of dwc:Taxon information (e.g. Lycosidae034_GHA).
sex	The sex of the biological individual (e.g. female).
measurementRemarks	Comments or notes accompanying the measurements (e.g. "detached opisthosoma").
Total body length mm	Maximum Opisthosoma length + Maximum Prosoma length in millimetres (excl. chelicerae and spinnerets).
Prosoma length mm	Maximum length of the prosoma antero-posteriorly from clypeus to pedicel in millimetres.
Prosoma width mm	Maximum length of the prosoma meso-laterally in millimetres.
Prosoma height mm	Maximum length of the prosoma dorso-ventrally in millimetres.
Tibia I length mm	Maximum length of the outer tibia I (leg) in millimetres.
Fang length mm	Maximum length of the fang from outer base to tip in millimetres.
measurementMethod	A description of the method used to determine the measurement (e.g. microscopy).

### Data set 3.

#### Data set name

Morphological measurements of ants (Hymenoptera, Formicidae) collected from northern Ghanaian mango orchards and forest savannah

#### Download URL

https://datadryad.org/stash/dataset/doi: 10.5061/dryad.h18931zwc

#### Description

This dataset contains the values of morphological traits measured from ants collected in northern Ghana October/November 2022. The four measured morphological traits for ants are Total body length (mm), Head length (mm), Scape length (mm) and Eye distance (mm).

**Data set 3.** 

Column label	Column description
organismID	An identifier for the dwc:Organism instance.
subfamily	The full scientific name of the subfamily in which the dwc:Taxon is classified (e.g. Formicinae).
genus	The full scientific name of the genus in which the dwc:Taxon is classified (e.g. Camponotus).
scientificName	The full scientific name, with authorship and date information if known (e.g. *Camponotus sericeus* (Fabricius, 1798)).
taxonRank	The taxonomic rank of the most specific name in the dwc:scientificName (e.g. subspecies).
identificationID	An identifier for the set of dwc:Taxon information (e.g. Pheidole002_GHA).
caste	Categorisation of individuals for eusocial species (e.g. worker).
measurementRemarks	Comments or notes accompanying the measurements (e.g. "head dented").
Total body length mm	Maximum Head length + Maximum Mesosomal (Weber's) length + Maximum Metasomal length in millimetres.
Head length mm	Maximum length of the head excluding mandibles in millimetres.
Scape length mm	Maximum length of the 1^st^ antennal segment.
Eye distance mm	Minimum length of the distance between compound eye and mandibular base in millimetres.
measurementMethod	A description of the method used to determine the measurement (e.g. microscopy).

## Additional information

Our survey revealed 64 (morpho)species of spiders from 47 genera and 22 families (Table 1). The total sample size was 438 individuals, with 291 males and 147 females (juveniles were not counted). In total, 36 taxa (56%) were identified to species level and 28 (44%) to morphospecies. Approximately 40% of the observed taxa were singletons (only one occurrence), while the most abundant (morpho)species with 64 individuals was *Dusmadiores* sp. (Zodariidae009_GHA).

Our survey revealed 64 (morpho)species of ants from 27 genera and five subfamilies (Table 2). The total sample size was 7,849 individuals, of which four were males. In total, 40 taxa (62.5%) were identified to species level and 24 (37.5%) to morphospecies. Approximately 17% of the observed taxa were singletons (only one occurence), while the most abundant (morpho)species with over 2,700 individuals was *Pheidole* sp. (Pheidole002_GHA).

In Kumbungu mango orchards, species richness (mean 8.4 ± sd 2.19), Hill number 1; i.e. exponential Shannon entropy (6.5 ± 2.52) and evenness (0.907 ± 0.054) of spiders were rather low and variable (Table 3). The single plot in the Dallung mango orchard also revealed low values for species richness (8.0), Hill 1 (6.5) and evenness (0.901). Finally, in Sinsablegbinni Forest Reserve, species richness (10.0 ± 2.15), Hill 1 (8.6 ± 2.47) and evenness (0.947 ± 0.015) of spiders were higher on average.

In Kumbungu mango orchards, species richness (mean 14.7 ± sd 3.62), Hill 1 (6.6 ± 2.37) and evenness (0.918 ± 0.023) of ants were surprisingly high, but had high variance between plots (Table 3). As with spiders, the single plot at Dallung mango orchard showed low values for species richness (10.7), Hill 1 (6.1) and evenness (0.894) of ants. Finally, in Sinsablegbinni Forest Reserve, species richness (14.7 ± 2.13), Hill 1 (5.57 ± 1.45) and evenness (0.935 ± 0.015) of ants were more or less the same as in Kumbungu.

In addition to taxonomic alpha diversity, we wanted to add a functional dimension. Given the time and resource constraints of this study, only morphological measures were taken. The averaged measures (mean) of morphological traits of each species are represented for spiders (Table 4) and ants (Table 5), respectively.

Finally, we calculated the community weighted means of each spider (Table 6) and ant trait (Table 7). While the Community Weighted Mean (CWM) of a trait has been criticised due to its tendency to be overly optimistic when testing for trait-environment relationships, it is still a powerful tool to understand both effect and response traits in ecosystem research (Lepš and de Bello 2023). We calculated CWMs for all traits across plots to provide a first insight into the differences in trait diversity that could drive ecosystem functioning.

## Figures and Tables

**Table 1.** List of all spider (morpho)species in taxonomic and alphabetical order by their family, the first records from Ghana, presence/absence within habitats and the total number of observed individuals.

**Species/morphospecies**	**Family**	**First record from Ghana**	**Mango**	**Savannah**	**Abundance**
*Larinia* sp. Araneidae027_GHA	Araneidae			X	1
*Neoscona blondeli* (Simon, 1886)	Araneidae	no	X		1
*Neoscona cereolella* (Strand, 1907)	Araneidae	yes	X		1
*Clubiona* sp. Clubionidae037_GHA	Clubionidae		X		1
*Cambalida compressa* Haddad, 2012	Corinnidae	yes		X	1
*Cambalida fulvipes* (Simon, 1896)	Corinnidae	yes	X		1
*Anahita aculeata* (Simon, 1897)	Ctenidae	yes	X	X	4
*Acontius* sp. Cyrtaucheniidae024_GHA	Cyrtaucheniidae		X	X	2
*Hongkongia* sp. Gnaphosidae006_GHA	Gnaphosidae		X	X	5
*Minosia clypeolaria* (Simon, 1907)	Gnaphosidae	yes	X		28
*Minosia eburneensis* Jézéquel, 1965	Gnaphosidae	yes	X		2
*Synaphosus yatenga* Ovtsharenko, Levy & Platnick, 1994	Gnaphosidae	yes		X	1
*Zelotes cassinensis* FitzPatrick, 2007	Gnaphosidae	yes	X		7
*Zelotes scrutatus* (O.Pickard-Cambridge, 1872)	Gnaphosidae	yes		X	2
*Agyneta prosectes* (Locket, 1968)	Linyphiidae	yes		X	1
*Ceratinopsis idanrensis* Locket & Russell-Smith, 1980	Linyphiidae	yes	X		5
*Ceratinopsis machadoi* (Miller, 1970)	Linyphiidae	yes		X	7
*Erigone prominens* Bösenberg & Strand, 1906	Linyphiidae	yes	X		19
*Metaleptyphantes perexiguus* (Simon & Fage, 1922)	Linyphiidae	yes	X	X	3
*Amblyothele hamatula* Russell-Smith, Jocqué & Alderweireldt, 2009	Lycosidae	yes		X	1
*Amblyothele* sp. Lycosidae023_GHA	Lycosidae			X	22
*Arctosa* sp. Lycosidae034_GHA	Lycosidae		X		2
*Foveosa albicapillis* Russell-Smith, Alderweireldt & Jocqué, 2007	Lycosidae	yes	X	X	7
Lycosidae030_GHA	Lycosidae		X		1
*Pardosa* sp. Lycosidae033_GHA	Lycosidae		X		1
*Trochosa mundamea* Roewer, 1960	Lycosidae	yes	X	X	6
*Trochosa* sp. Lycosidae010_GHA	Lycosidae			X	5
*Trochosa* sp. Lycosidae029_GHA	Lycosidae		X		34
*Speocera* sp. Ochyroceratidae014_GHA	Ochyroceratidae			X	6
*Oecobius* sp. Oecobiidae005_GHA	Oecobiidae		X	X	27
*Antoonops kamieli* Fannes, 2013	Oonopidae	yes	X	X	3
Oonopidae020_GHA	Oonopidae		X	X	21
Oonopidae026_GHA	Oonopidae			X	1
*Opopaea* sp. Oonopidae036_GHA	Oonopidae		X		3
*Oxyopes dumonti* (Vinson, 1863)	Oxyopidae	yes		X	3
*Oxyopes* sp. Oxyopidae021_GHA	Oxyopidae			X	1
*Oxyopes* sp. Oxyopidae028_GHA	Oxyopidae		X		1
*Oxyopes* sp. Oxyopidae032_GHA	Oxyopidae		X		1
*Scelidocteus* sp. Palpimanidae015_GHA	Palpimanidae			X	1
*Thanatus* sp. Philodromidae038_GHA	Philodromidae		X		8
*Tibellus minor* Lessert, 1919	Philodromidae	yes	X		4
*Perenethis simoni* (Lessert, 1916)	Pisauridae	yes	X		1
*Evarcha idanrensis* Wesołowska & Russell-Smith, 2011	Salticidae	yes		X	2
*Hyllus dotatus* (G. W. Peckham & E. G. Peckham, 1903)	Salticidae	yes	X		1
*Langelurillus quadrimaculatus* Wesolowska & Russel-Smith, 2011	Salticidae	no	X	X	9
*Langona bristowei* Berland & Millot, 1941	Salticidae	yes	X		1
*Menemerus eburnensis* Berland & Millot, 1941	Salticidae	yes		X	1
*Myrmarachne kiboschensis* Lessert, 1925	Salticidae	yes	X		2
*Phlegra pusilla* Wesołowska & van Harten, 1994	Salticidae	yes	X	X	2
*Phlegra touba* Logunov & Azarkina, 2006	Salticidae	yes	X	X	15
*Stenaelurillus wa* Wawer & Wesolowska, 2025	Salticidae	no		X	6
Salticidae102_GHA	Salticidae		X		1
*Scytodes reticulata* Jézéquel, 1964	Scytodidae	yes		X	1
*Cepheia longiseta* (Simon, 1881)	Synaphridae	yes	X		1
*Tetragnatha jaculator* Tullgren, 1910	Tetragnathidae	yes	X		1
*Argyrodes argyrodes* (Walckenaer, 1841)	Theridiidae	yes	X		1
Theridiidae013_GHA	Theridiidae			X	1
*Bassaniodes* sp. Thomisidae035_GHA	Thomisidae		X		12
*Ozyptila* sp. Thomisidae008_GHA	Thomisidae			X	2
*Acanthinozodium sahelense* Jocqué & Henrard, 2015	Zodariidae	yes		X	7
*Dusmadiores* sp. Zodariidae009_GHA	Zodariidae		X	X	64
*Mallinella* sp. Zodariidae022_GHA	Zodariidae		X	X	9
*Mallinella* sp. Zodariidae031_GHA	Zodariidae		X		4
Zodariidae012_GHA	Zodariidae			X	42

**Table 2.** List of all ant (morpho)species in taxonomic and alphabetical order by their subfamily, the first records from Ghana, presence/absence within habitats and the total number of observed individuals.

**Species/morphospecies**	**Subfamily**	**First record from Ghana**	**Mango**	**Savannah**	**Abundance**
*Tapinoma carininotum* Weber, 1943	Dolichoderinae	yes	X	X	14
*Aenictus boltoni* Gomez, 2022	Dorylinae	no	X		5
*Aenictus guineensis* Santschi, 1924	Dorylinae	no	X		1
*Dorylus braunsi* Emery, 1895	Dorylinae	yes		X	77
*Dorylus spininodis* Emery, 1901	Dorylinae	no	X	X	1067
*Parasyscia kenyensis* (Consani, 1951)	Dorylinae	yes		X	1
*Parasyscia sudanensis* (Weber, 1942)	Dorylinae	no		X	1
Agraulomyrmex001_GHA	Formicinae	yes		X	1
*Camponotus carbo occidentalis* Mayr, 1902	Formicinae	no	X	X	36
*Camponotus maculatus* species complex	Formicinae		X	X	189
*Camponotus sericeus* (Fabricius, 1798)	Formicinae	no	X	X	101
Camponotus001_GHA	Formicinae		X	X	7
Camponotus002_GHA	Formicinae			X	78
Camponotus003_GHA	Formicinae			X	7
Camponotus004_GHA	Formicinae			X	63
Camponotus005_GHA	Formicinae			X	24
*Lepisiota capensis guineensis* (Mayr, 1902)	Formicinae	no	X	X	287
*Lepisiota capensis laevis* (Santschi, 1913)	Formicinae	no	X	X	71
Lepisiota001_GHA	Formicinae		X		1
Lepisiota002_GHA	Formicinae		X		4
*Nylanderia scintilla* LaPolla & Fisher, 2011	Formicinae	yes	X		37
*Oecophylla longinoda* (Latreille, 1802)	Formicinae	no	X		45
*Paratrechina longicornis* (Latreille, 1802)	Formicinae	no	X		2
*Polyrhachis viscosa* Smith, F., 1858	Formicinae	no		X	4
*Cardiocondyla emeryi* Forel, 1881	Myrmicinae	no	X		2
*Cardiocondyla yoruba* Rigato, 2002	Myrmicinae	no	X	X	3
Carebara001_GHA	Myrmicinae		X		12
Crematogaster001_GHA	Myrmicinae		X	X	18
Crematogaster002_GHA	Myrmicinae		X	X	33
Crematogaster003_GHA	Myrmicinae		X		1
Crematogaster004_GHA	Myrmicinae			X	5
*Meranoplus magrettii* André, 1884	Myrmicinae	no		X	60
*Monomorium afrum* André, 1884	Myrmicinae	no		X	9
*Monomorium balathir* Bolton, 1987	Myrmicinae	no	X	X	23
*Monomorium bicolor* Emery, 1877	Myrmicinae	no	X	X	144
*Monomorium mictilis* Forel, 1910	Myrmicinae	yes	X	X	7
Monomorium001_GHA	Myrmicinae		X	X	53
Monomorium002_GHA	Myrmicinae		X		3
*Myrmicaria salambo* Wheeler, W.M., 1922	Myrmicinae	no	X	X	969
Myrmicaria001_GHA	Myrmicinae			X	2
Pheidole001_GHA	Myrmicinae			X	319
Pheidole002_GHA	Myrmicinae		X	X	2734
Pheidole003_GHA	Myrmicinae		X	X	187
Pheidole004_GHA	Myrmicinae		X	X	419
Pheidole005_GHA	Myrmicinae		X	X	11
*Strumigenys rufobrunea* Santschi, 1914	Myrmicinae	no	X	X	2
*Tetramorium angulinode* Santschi, 1910	Myrmicinae	no		X	150
*Tetramorium anxium* Santschi, 1914	Myrmicinae	no	X		10
*Tetramorium caldarium* (Roger, 1857)	Myrmicinae	yes	X	X	37
*Tetramorium calinum* Bolton, 1980	Myrmicinae	no		X	12
*Tetramorium dysderke* Bolton, 1980	Myrmicinae	yes	X		1
*Tetramorium ericae* Arnold, 1917	Myrmicinae	yes		X	12
*Tetramorium sericeiventre* Emery, 1877	Myrmicinae	no		X	1
*Tetramorium zapyrum* Bolton, 1980	Myrmicinae	no	X	X	54
Tetramorium001_GHA	Myrmicinae			X	1
*Trichomyrmex abyssinicus* (Forel, 1894)	Myrmicinae	no		X	67
*Trichomyrmex oscaris* (Forel, 1894)	Myrmicinae	no	X	X	59
*Bothroponera silvestrii* Santschi, 1914	Ponerinae	no	X	X	9
*Bothroponera soror* (Emery, 1899)	Ponerinae	no		X	197
*Brachyponera sennaarensis* (Mayr, 1862)	Ponerinae	no	X		1
*Hypoponera punctatissima* (Roger, 1859)	Ponerinae	no	X		2
*Leptogenys longiceps* Santschi, 1914	Ponerinae	no		X	3
*Odontomachus troglodytes* Santschi, 1914	Ponerinae	no	X		93
*Plectroctena macgeei* Bolton, 1974	Ponerinae	no	X		1

**Table 3.** Alpha diversity of spiders and ants within each plot. Observed mean species richness, the exponent of Shannon diversity (Hill q = 1) and evenness were rarefied and calculated with R package 'BAT' 2.9.6. (Cardoso et al. 2015).

**Spiders**				**Ants**		
**Plot**	**Species richness**	**Hill q = 1**	**Evenness**	**Species richness**	**Hill q = 1**	**Evenness**
**Kumbungu 1**	11.79	10.378	0.959	16.64	8.196	0.932
**Kumbungu 2**	8.59	5.638	0.914	13.67	5.504	0.923
**Kumbungu 3**	5.80	3.449	0.818	9.16	3.262	0.884
**Kumbungu 4**	8.40	6.858	0.940	18.81	9.358	0.943
**Kumbungu 5**	7.43	6.207	0.904	15.00	6.568	0.906
**Dallung**	8.00	6.492	0.901	10.74	6.112	0.894
**Sinsablegbinni 1**	12.85	11.742	0.969	13.86	5.956	0.930
**Sinsablegbinni 2**	10.52	9.340	0.947	12.97	3.684	0.922
**Sinsablegbinni 3**	8.15	6.474	0.941	14.29	5.460	0.932
**Sinsablegbinni 4**	8.57	6.784	0.932	17.83	7.172	0.956

**Table 4.** The average of each morphological trait by spider (morpho)species. TL = total body length, PrL = prosoma length, PrW = prosoma width, PrH = prosoma height, TIL = tibia I length, FL = fang length. All measures in millimetres. Values are rounded to three decimals. Missing values are marked as NAs.

**(Morpho)species**	**n**	**TL**	**PrL**	**PrW**	**PrH**	**TIL**	**FL**
*Larinia* sp. Araneidae027_GHA	1	3.100	1.600	1.150	0.750	2.000	0.250
*Neoscona blondeli* (Simon, 1886)	1	9.500	3.600	3.000	1.500	NA	0.800
*Neoscona cereolella* (Strand, 1907)	1	4.400	2.250	1.750	1.250	1.750	0.338
*Clubiona* sp. Clubionidae037_GHA	1	4.100	1.950	1.350	0.850	0.900	0.450
*Cambalida compressa* Haddad, 2012	1	6.100	2.850	2.000	1.600	2.100	0.680
*Cambalida fulvipes* (Simon, 1896)	1	NA	1.875	1.350	0.975	NA	0.388
*Anahita aculeata* (Simon, 1897)	4	10.035	4.868	3.539	1.592	4.272	1.134
*Acontius* sp. Cyrtaucheniidae024_GHA	2	6.762	2.839	1.897	2.000	1.351	0.959
*Hongkongia* sp. Gnaphosidae006_GHA	5	5.444	2.295	1.696	1.010	1.563	0.444
Minosia clypeolaria (Simon, 1907)	5	6.148	3.079	2.427	1.785	1.631	0.416
*Minosia eburneensis* Jézéquel, 1965	2	4.952	2.348	1.687	1.196	1.549	0.354
*Synaphosus yatenga* Ovtsharenko, Levy & Platnick, 1994	1	2.575	1.225	0.875	0.560	0.675	0.125
*Zelotes scrutatus* (O.Pickard-Cambridge, 1872)	2	5.294	2.372	1.749	0.995	1.299	0.618
*Zelotes cassinensis* FitzPatrick, 2007	7	7.241	3.439	2.611	1.599	1.826	0.589
*Agyneta prosectes* (Locket, 1968)	1	1.380	0.560	0.400	0.375	0.525	0.100
*Ceratinopsis idanrensis* Locket & Russell-Smith, 1980	5	1.131	0.625	0.502	0.407	0.437	0.130
*Ceratinopsis machadoi* (Miller, 1970)	6	1.252	0.610	0.516	0.401	0.444	0.141
*Metaleptyphantes perexiguus* (Simon & Fage, 1922)	10	1.170	0.521	0.409	0.317	0.482	0.097
*Erigone prominens* Bösenberg & Strand, 1906	3	1.203	0.649	0.532	0.524	0.497	0.178
*Amblyothele* sp. Lycosidae023_GHA	1	4.400	2.175	1.700	1.150	1.250	0.375
*Amblyothele hamatula* Russell-Smith, Jocqué & Alderweireldt, 2009	10	2.763	1.336	1.036	0.748	1.207	0.227
*Arctosa* sp. Lycosidae034_GHA	2	4.469	2.510	1.871	1.500	1.150	0.530
*Foveosa albicapillis* Russell-Smith, Alderweireldt & Jocqué, 2007	5	2.527	1.408	1.099	0.968	0.998	0.216
Lycosidae030_GHA	1	7.700	4.100	3.000	2.500	3.200	0.900
*Pardosa* sp. Lycosidae033_GHA	1	5.500	2.800	2.100	1.600	1.675	0.600
*Trochosa* sp. Lycosidae010_GHA	5	10.242	5.258	3.749	3.045	2.808	1.123
*Trochosa mundamea* Roewer, 1960	5	11.345	5.566	4.214	3.517	2.932	1.276
*Trochosa* sp. Lycosidae029_GHA	10	5.729	3.150	2.401	1.716	1.903	0.606
*Speocera* sp. Ochyroceratidae014_GHA	6	1.091	0.485	0.408	0.320	0.694	0.098
*Oecobius* sp. Oecobiidae005_GHA	10	1.635	0.640	0.733	0.537	0.510	0.060
*Antoonops kamieli* Fannes, 2013	3	1.437	0.754	0.559	0.398	0.249	0.091
Oonopidae020_GHA	10	1.421	0.724	0.524	0.441	0.380	0.099
Oonopidae026_GHA	1	2.025	0.875	0.700	0.500	0.650	0.150
*Opopaea* sp. Oonopidae036_GHA	3	1.571	0.650	0.517	0.385	0.254	0.083
*Oxyopes dumonti* (Vinson, 1863)	3	3.777	1.830	1.299	0.909	1.872	0.258
*Oxyopes* sp. Oxyopidae021_GHA	1	NA	1.550	1.250	0.650	1.875	0.213
*Oxyopes* sp. Oxyopidae028_GHA	1	4.750	2.000	1.500	1.300	2.100	0.275
*Oxyopes* sp. Oxyopidae032_GHA	1	3.420	1.750	1.250	1.000	1.350	0.200
*Scelidocteus* sp. Palpimanidae015_GHA	1	4.850	2.200	1.500	1.400	0.900	0.288
*Tibellus minor* Lessert, 1919	4	8.857	3.409	2.334	1.409	4.652	0.405
*Thanatus* sp. Philodromidae038_GHA	6	4.563	2.505	2.523	1.555	2.758	0.298
*Perenethis simoni* (Lessert, 1916)	1	10.800	3.500	2.750	1.500	6.000	0.900
*Evarcha idanrensis* Wesołowska & Russell-Smith, 2011	2	5.144	2.225	1.686	1.500	0.762	0.331
*Hyllus dotatus* (G. W. Peckham & E. G. Peckham, 1903)	1	4.700	2.000	1.350	1.750	1.500	0.475
*Langelurillus quadrimaculatus* Wesolowska & Russel-Smith, 2011	6	3.614	1.784	1.384	1.239	0.511	0.194
*Langona bristowei* Berland & Millot, 1941	1	7.000	3.500	2.450	2.300	0.900	0.438
*Menemerus eburnensis* Berland & Millot, 1941	1	4.350	2.200	1.600	0.850	0.850	0.525
*Myrmarachne kiboschensis* Lessert, 1925	2	5.518	2.325	1.275	1.049	1.373	0.788
*Phlegra pusilla* Wesołowska & van Harten, 1994	2	2.324	1.186	0.776	0.704	0.374	0.149
*Phlegra touba* Logunov & Azarkina, 2006	10	3.063	1.534	1.068	0.898	0.422	0.201
*Stenaelurillus wa* Wawer & Wesolowska, 2025	6	4.812	2.384	1.706	1.574	0.691	0.271
Salticidae102_GHA	1	3.250	1.750	1.300	1.300	0.580	0.250
*Scytodes reticulata* Jézéquel, 1964	1	3.600	1.750	1.350	1.000	NA	0.100
*Cepheia longiseta* (Simon, 1881)	1	0.900	0.300	0.310	0.300	0.240	0.075
*Tetragnatha jaculator* Tullgren, 1910	1	5.500	1.850	1.150	0.800	5.000	0.875
*Argyrodes argyrodes* (Walckenaer, 1841)	1	2.250	1.250	0.575	0.875	1.650	0.213
Theridiidae013_GHA	1	NA	1.500	1.300	0.875	1.375	0.250
*Bassaniodes* sp. Thomisidae035_GHA	6	4.898	2.518	2.442	1.354	1.730	0.288
*Ozyptila* sp. Thomisidae008_GHA	2	3.491	1.620	1.799	1.000	1.000	0.194
*Acanthinozodium sahelense* Jocqué & Henrard, 2015	5	3.488	1.823	1.371	1.007	1.845	0.145
*Dusmadiores* sp. Zodariidae009_GHA	10	1.705	0.832	0.595	0.483	0.504	0.063
*Mallinella* sp. Zodariidae022_GHA	8	6.843	3.423	2.343	1.947	1.930	0.370
*Mallinella* sp. Zodariidae031_GHA	4	6.487	3.181	2.120	1.680	1.598	0.323
Zodariidae012_GHA	10	2.417	1.222	0.963	0.767	1.232	0.108

**Table 5.** The average of each morphological trait by ant (morpho)species. TL = total body length, HL = head length, SL = scape length, ED = eye distance (from the mandibular base). All measures are in millimetres. Values are rounded to three decimals. Missing values are marked as NAs.

**(Morpho)species**	**n**	**TL**	**HL**	**SL**	**ED**
*Tapinoma carininotum* Weber, 1943	5	1.871	0.452	0.407	0.092
*Aenictus boltoni* Gomez, 2022	4	2.580	0.569	0.307	NA
*Aenictus guineensis* Santschi, 1924	1	3.500	0.675	0.488	NA
*Dorylus braunsi* Emery, 1895	5	3.901	0.962	0.443	NA
*Dorylus spininodis* Emery, 1901	5	5.347	1.094	0.433	NA
*Parasyscia kenyensis* (Consani, 1951)	1	3.313	0.675	0.350	0.275
*Parasyscia sudanensis* (Weber, 1942)	1	2.975	0.575	0.313	0.213
Agraulomyrmex001_GHA	1	1.163	0.300	0.225	0.050
*Camponotus carbo occidentalis* Mayr, 1902	5	7.003	1.533	1.874	0.746
*Camponotus maculatus* species complex	7	9.324	2.046	2.499	0.888
*Camponotus sericeus* (Fabricius, 1798)	5	8.292	1.805	1.748	0.815
Camponotus001_GHA	5	8.986	2.128	2.005	1.024
Camponotus002_GHA	5	7.384	1.546	2.125	0.746
Camponotus003_GHA	5	7.703	1.570	2.008	0.638
Camponotus004_GHA	5	8.581	1.767	2.346	0.838
Camponotus005_GHA	5	6.614	1.326	1.354	0.663
*Lepisiota capensis guineensis* (Mayr, 1902)	5	2.925	0.602	0.727	0.165
*Lepisiota capensis laevis* (Santschi, 1913)	5	1.747	0.425	0.462	0.105
Lepisiota001_GHA	1	1.725	0.425	0.450	0.150
Lepisiota002_GHA	4	2.664	0.559	0.721	0.193
*Nylanderia scintilla* LaPolla & Fisher, 2011	5	1.962	0.505	0.575	0.125
*Oecophylla longinoda* (Latreille, 1802)	5	7.679	1.601	2.250	0.504
*Paratrechina longicornis* (Latreille, 1802)	2	2.760	0.643	1.175	0.194
*Polyrhachis viscosa* Smith, F., 1858	3	7.668	1.638	1.947	0.732
*Cardiocondyla emeryi* Forel, 1881	2	1.681	0.425	0.288	0.050
*Cardiocondyla yoruba* Rigato, 2002	3	1.650	0.421	0.267	0.071
Carebara001_GHA	6	1.398	0.374	0.223	0.097
Crematogaster001_GHA	5	3.802	0.810	0.637	0.319
Crematogaster002_GHA	5	3.113	0.660	0.560	0.237
Crematogaster003_GHA	1	3.213	0.663	0.513	0.263
Crematogaster004_GHA	4	1.924	0.450	0.300	0.181
*Meranoplus magrettii* André, 1884	5	3.141	0.699	0.515	0.230
*Monomorium afrum* André, 1884	5	4.112	0.905	0.771	0.252
*Monomorium balathir* Bolton, 1987	5	2.014	0.477	0.342	0.088
*Monomorium bicolor* Emery, 1877	5	3.207	0.694	0.617	0.185
*Monomorium mictilis* Forel, 1910	5	1.533	0.352	0.212	0.070
Monomorium001_GHA	5	1.575	0.372	0.252	0.077
Monomorium002_GHA	2	1.594	0.375	0.244	0.063
*Myrmicaria salambo* Wheeler, W.M., 1922	2	8.405	1.473	1.612	0.662
Myrmicaria001_GHA	5	8.894	1.648	1.760	0.692
Pheidole001_GHA	5	2.934	0.614	0.647	0.130
Pheidole002_GHA	5	3.447	0.765	1.055	0.177
Pheidole003_GHA	5	3.117	0.647	0.822	0.147
Pheidole004_GHA	5	2.417	0.510	0.472	0.110
Pheidole005_GHA	5	2.682	0.586	0.617	0.130
*Strumigenys rufobrunea* Santschi, 1914	2	2.199	0.525	0.275	0.219
*Tetramorium angulinode* Santschi, 1910	5	2.553	0.567	0.347	0.127
*Tetramorium anxium* Santschi, 1914	5	2.263	0.517	0.365	0.117
*Tetramorium caldarium* (Roger, 1857)	5	2.232	0.492	0.352	0.110
*Tetramorium calinum* Bolton, 1980	5	3.594	0.767	0.522	0.205
*Tetramorium dysderke* Bolton, 1980	1	3.040	0.600	0.438	0.213
*Tetramorium ericae* Arnold, 1917	5	1.977	0.460	0.290	0.090
*Tetramorium sericeiventre* Emery, 1877	1	3.500	0.813	0.775	0.256
*Tetramorium zapyrum* Bolton, 1980	5	3.047	0.657	0.427	0.190
Tetramorium001_GHA	1	4.360	0.860	0.640	0.200
*Trichomyrmex abyssinicus* (Forel, 1894)	5	4.193	1.084	0.731	0.264
*Trichomyrmex oscaris* (Forel, 1894)	5	2.081	0.507	0.357	0.115
*Bothroponera silvestrii* Santschi, 1914	5	5.883	1.135	0.890	0.230
*Bothroponera soror* (Emery, 1899)	5	8.907	1.595	1.285	0.335
*Hypoponera punctatissima* (Roger, 1859)	1	2.740	0.550	0.363	0.063
*Leptogenys longiceps* Santschi, 1914	3	4.875	0.875	0.807	0.142
*Odontomachus troglodytes* Santschi, 1914	5	10.559	2.510	2.270	0.529
*Plectroctena macgeei* Bolton, 1974	1	12.650	2.500	1.550	0.300

**Table 6.** Community Weighted Mean (the average of the local distribution of a trait in a community) of spider traits within each plot. Calculated with R package 'BAT' 2.9.6. (Cardoso et al. 2015).

Spiders						
Plot	Total body length (mm)	Prosoma length (mm)	Prosoma width (mm)	Prosoma heigth (mm)	Tibia I length (mm)	Fang length (mm)
Kumbungu 1	5.929	2.621	1.865	1.383	2.181	0.405
Kumbungu 2	5.193	2.504	1.931	1.426	1.275	0.406
Kumbungu 3	4.688	2.287	1.774	1.305	1.442	0.355
Kumbungu 4	4.381	2.264	1.853	1.292	1.424	0.386
Kumbungu 5	3.361	1.726	1.440	1.044	1.236	0.250
Dallung	3.383	1.710	1.241	0.962	1.158	0.364
Sinsablegbinni 1	3.860	1.877	1.460	1.100	1.147	0.313
Sinsablegbinni 2	2.631	1.254	0.972	0.745	0.823	0.173
Sinsablegbinni 3	2.485	1.217	0.915	0.715	0.884	0.157
Sinsablegbinni 4	2.792	1.430	1.047	0.818	0.990	0.189

**Table 7.** Community Weighted Mean (the average of the local distribution of a trait in a community) of ant traits within each plot. Calculated with R package 'BAT' 2.9.6. (Cardoso et al. 2015).

Ants				
Plot	Total body length (mm)	Head length (mm)	Scape length (mm)	Eye distance (mm)
Kumbungu 1	4.947	1.090	1.166	0.340
Kumbungu 2	6.821	1.343	1.295	0.426
Kumbungu 3	7.035	1.333	1.418	0.517
Kumbungu 4	3.835	0.849	0.970	0.244
Kumbungu 5	4.107	0.877	0.735	0.143
Dallung	5.015	1.129	1.185	0.274
Sinsablegbinni 1	5.112	1.043	1.171	0.334
Sinsablegbinni 2	3.702	0.817	0.991	0.203
Sinsablegbinni 3	4.409	0.925	1.028	0.233
Sinsablegbinni 4	4.187	0.891	0.789	0.156
